# Association of Vasculitis and Familial Mediterranean Fever

**DOI:** 10.3389/fimmu.2019.00763

**Published:** 2019-04-12

**Authors:** Salam Abbara, Gilles Grateau, Stéphanie Ducharme-Bénard, David Saadoun, Sophie Georgin-Lavialle

**Affiliations:** ^1^Sorbonne Université, INSERM UMRS_933, AP-HP, Hôpital Tenon, Service de Médecine Interne, Centre de Référence des Maladies Auto-Inflammatoires et des Amyloses d'Origine Inflammatoire (CEREMAIA), Paris, France; ^2^Sorbonne Université, AP-HP, Hôpital Pitié-Salpêtrière, Service de Médecine Interne, Centre de Référence des Maladies Auto-Inflammatoires et des Amyloses d'Origine Inflammatoire (CEREMAIA), Paris, France

**Keywords:** familial Mediterranean fever, vasculitis, autoinflammatory syndrome, *MEFV*, Behçet disease

## Abstract

Certain types of vasculitis occur more frequently and present differently in patients with familial Mediterranean fever (FMF). We assessed the characteristics of patients with FMF and systemic vasculitis through a systematic review of the literature. Medline was searched by two independent investigators until December 2017. We screened 310 articles and selected 58 of them (IgA vasculitis *n* = 12, polyarteritis nodosa (PAN) *n* = 25, Behçet's disease (BD) *n* = 7, other vasculitis *n* = 14). Clinical case reports were available for 167 patients (IgA vasculitis *n* = 46, PAN *n* = 61, BD *n* = 46, other vasculitis *n* = 14), and unavailable for 45 patients (IgA vasculitis *n* = 38, PAN *n* = 7). IgA vasculitis was the most common vasculitis in FMF patients with a prevalence of 2.7–7%, followed by PAN with a prevalence of 0.9–1.4%. Characteristics of FMF did not differ between patients with and without vasculitis. Patients with FMF and IgA vasculitis displayed more intussusception (8.7%) and possibly less IgA deposits on histological analysis than patients with IgA vasculitis alone. Patients with FMF and PAN had a younger age at vasculitis onset (mean age = 17.9 years), as well as more perirenal hematomas (49%) and CNS involvement (31%) than patients with PAN alone. Glomerular involvement was noted in 33% of patients diagnosed with PAN, suggesting an alternative diagnosis. Sequencing of the *MEFV* gene confirmed the presence of two pathogenic variants in 73% of FMF patients with IgA vasculitis or PAN. The majority of patients with BD were from one case series, and presented more skin, gastrointestinal, and CNS involvement than patients with isolated BD. In conclusion, FMF, particularly when supported by two pathogenic *MEFV* mutations, could predispose to IgA vasculitis, or a PAN-like vasculitis with more perirenal bleeding and CNS involvement.

## Introduction

Familial Mediterranean fever (FMF) is the most common hereditary autoinflammatory disease, usually affecting people of Mediterranean descent (1). It is caused by autosomal recessive inheritance of mutations in the Mediterranean fever (*MEFV*) gene (1). p.Met694Val is the most frequently involved mutation, although the pathogenicity of other variants such as V726A and M694I has now been established. The majority of these pathogenic variants are located in exon 10. Neutrophils and monocytes express pyrin, a protein encoded by *MEFV*, which regulates innate immunity through an inflammasome that leads to the production of interleukin (IL)-1 beta (2). Vasculitis are a group of diseases characterized by inflammation of blood vessels. Several retrospective studies have reported an increased incidence of vasculitis in FMF patients, namely IgA vasculitis and polyarteritis nodosa (PAN) (3–6). IgA vasculitis, which primarily affects children, is characterized by deposition of IgA-containing immune complexes in the wall of small vessels. It has been described in 2.7–7% of FMF patients residing in Turkey and Israel (3–5). Polyarteritis nodosa (PAN), a necrotizing vasculitis affecting medium-sized arteries, was reported in 0.9–1.4% of FMF patients from these same countries (4, 5). Conversely, in a single retrospective study of 4,000 FMF patients, only 0.4% developed Behçet's disease (6). Case reports of FMF patients with unclassified vasculitis of variable severity have also been published, suggesting the existence of a distinct FMF-related vasculitis. In a previous review, Aksu et al. presented the clinical characteristics and pathophysiology underlying the association between FMF and vasculitis. More recently, Jain et al. analyzed 27 publications on this topic (7, 8). To our knowledge, a systematic analysis of all cases of FMF-associated vasculitis reported in the literature has never been completed. We therefore performed a systematic literature review on the co-occurrence of FMF and vasculitis, in order to define the characteristics of patients with both FMF and vasculitis.

## Methods

### Systematic Review

A systematic review of clinical studies published in the medical literature was conducted to retrieve all cases of FMF-associated vasculitis in both adults and children. MEDLINE was searched through PubMed until December 2017, with no language restriction or publication date limit. The search strategy included MESH terms, such as ((“Behcet Syndrome”[Mesh] AND “Vasculitis”[Mesh]) AND “Familial Mediterranean Fever”[Mesh]). Two independent investigators (SA, SGL) screened titles and abstracts to exclude those unrelated to the topic at hand. Remaining articles were assessed for eligibility. The reference lists of these articles were also searched for relevant titles. Only full-text articles were included. When a full-text article was not available online, it was searched for at the Inter-University Science Library of Paris or requested from the Center of medical-pharmaceutical documentation of the Public Assistance Hospitals of Paris. When these steps failed to provide the full-text article, corresponding authors were contacted by email. Full-text articles written in a language other than English or French were translated prior to analysis. Eligible papers for which full texts were unavailable were cited but excluded from the analysis. For every article, we checked which criteria were used for the diagnosis of vasculitis. When the latter were not explicitly cited in the article, clinical case descriptions were retrospectively assessed in order to confirm fulfillment of international criteria for the vasculitis commonly associated with FMF, namely the 1990 ACR criteria for PAN, the 1990 ACR and 2010 EULAR criteria for IgA vasculitis, and the 1990 International Study Group (ISG) criteria for BD (9–12). The Livneh criteria were the most commonly used diagnostic criteria for FMF in the articles reviewed (13).

The following data were recorded from the articles when available: diagnostic criteria for vasculitis, study population, number of cases of FMF-associated vasculitis, age, sex, ethnicity, family history, comorbid diseases, age at FMF and vasculitis onset, age at FMF and vasculitis diagnosis, presence or absence of *MEFV* mutation, age at colchicine initiation, mean colchicine dose, current colchicine dose, clinical presentation of FMF and vasculitis, history of treatment for FMF and vasculitis, activity of FMF under treatment based on symptoms and C-reactive protein, therapeutic response of vasculitis at last follow-up (remission, partial response, or death), presence or absence of amyloidosis, renal function, and variation of C-reactive protein, and serum amyloid A under treatment.

### Statistical Analysis

Patient characteristics were reported as means ± standard deviation for continuous variables, and as numbers (%) for categorical variables. Continuous variables were compared using the Student's *t*-test, and categorical variables were compared using the chi-square test or Fisher's exact test, when appropriate. All statistical analyses were performed using the online tool BiostaTGV. A 2-sided *p*-value < 0.05 was considered statistically significant.

## Results

**Figure 1:**
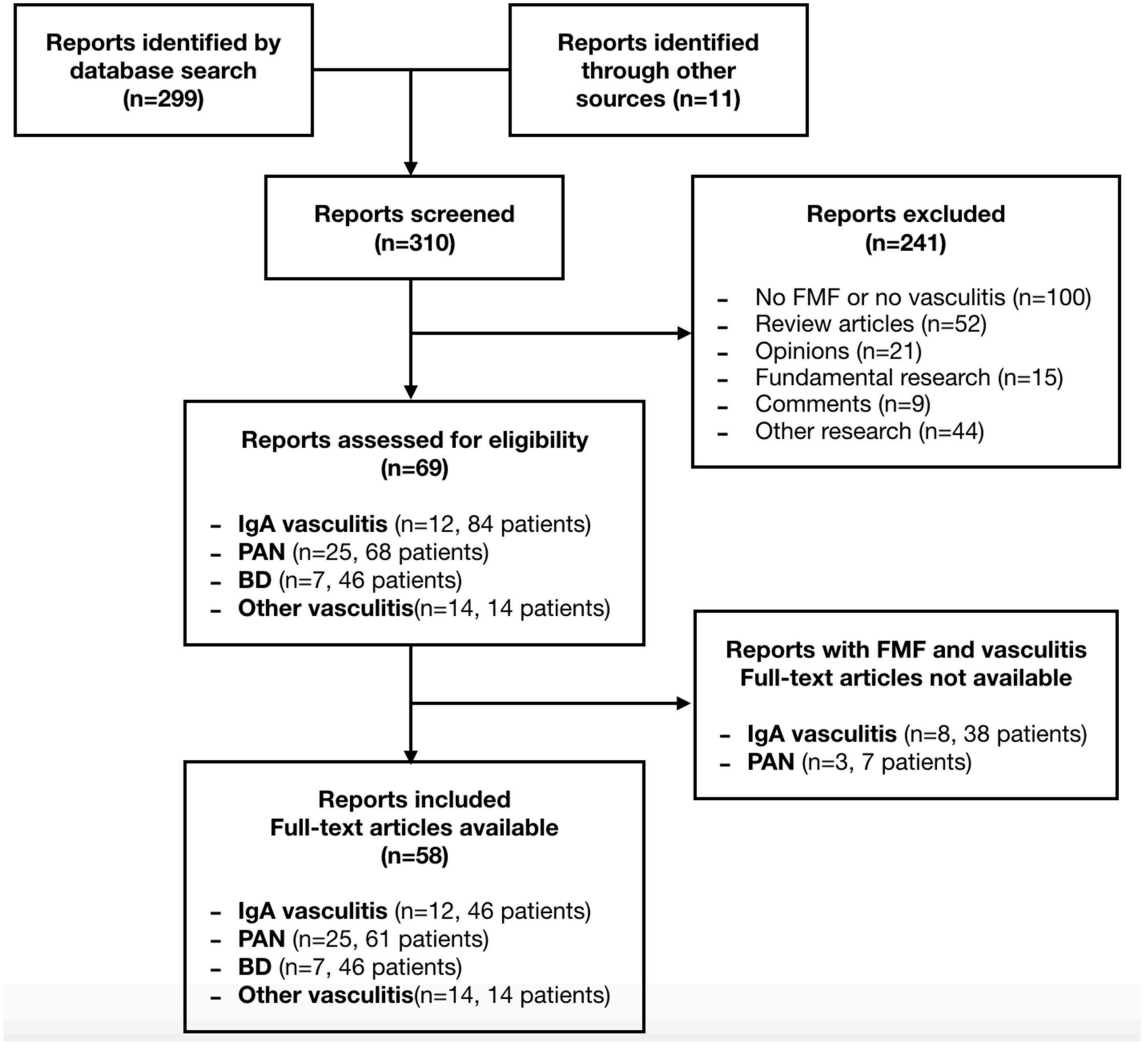
Flow diagram of the article selection process.

The literature search retrieved 310 articles. Sixty-nine articles were eligible based on their relevance to the topic. The full text of 11 articles was unavailable. In total, 58 articles were included. Two papers were not published in English (14, 15). The selection process is presented in the flow diagram (Figure 1). The main results are presented in Table 1.

**Table 1:** Characteristics of vasculitis in FMF patients compared to the general population.

**Vasculitis**	**Prevalence**	**Sex ratio men/women**	***MEFV*: percentage of two pathogenic mutations**	**Mean age at diagnosis/onset, years ± SD**	**Clinical and paraclinical characteristics**
IgA vasculitis	Increased (2.7–7%)	Unchanged 1.1 (*p* = 0.4278)	73.3% (11/15 patients)	Increased 10.5 ± 4.1	Increased intussusception (9%)
					Possibly less IgA deposits (23%)
PAN	Increased (0.9–1.4%)	Increased 3.6 (*p* = 0.012)	73.9% (17/23 patients)	Decreased 17.9 ± 8.5	Increased perirenal hematomas (49%) and CNS involvement (31%). Glomerular involvement in 33% of reports suggesting alternative diagnosis
BD	May be increased 0.4%	Decreased 0.4 (*p* = 0.0008)	33.3% (2/6 patients)	Similar 21.3 ± 4.0	Increased CNS (40.9%) involvement

*PAN, polyarteritis nodosa; BD, Behçet's disease; MPA, microscopic polyangiitis; CNS, central nervous system*.

### FMF and IgA Vasculitis (Tables 2, 3)

The annual incidence of IgA vasculitis in the general population varies from 3 to 26/100 000 children, and from 0.34 to 1.8/100 000 adults, depending on populations and studies (16, 17). The prevalence of IgA vasculitis in FMF patients was evaluated in four studies conducted in Turkey and Israel, with values ranging from 2.7 to 7.2% (4, 5, 18, 19). Only two studies compared the prevalence of IgA vasculitis in FMF patients to the prevalence in the general population. They found a prevalence of IgA vasculitis of, respectively, 3.6 and 7.2% in FMF patients, as compared to a prevalence of, respectively, 0.05 and 0.8% in controls from the same health centers (4, 5).

**Table 2:** Main clinical characteristics at diagnosis of the 46 patients with IgA vasculitis and FMF.

**Characteristics**	**Review of the literature, IgA vasculitis + FMF, *n* = 46**
**Number of men/women (ratio)**	21/20 (1.1)
**Ethnicity**, ***n*****(%)**	
Turkish	32 (69.6)
Jewish	12 (26.1)
Other	2 (4.3)
**FMF**	
Family history of FMF, *n*(%)	21 (45.7)
Age at diagnosis/onset, mean ± SD (years) @	6.8 ± 2.2
Genotyping of MEFV^**^	
**Two pathogenic variants**	11 (73.3)
M694V/M694V, *n*(%)	7 (46.6)
M694V/V726A, *n*(%)	3 (20.0)
M694V/M694I, *n*(%)	1 (6.7)
**One pathogenic variant**	4 (26.7)
M694V/-, *n*(%)	1 (6.7)
V726A/-, *n*(%)	2 (13.3)
M694I/-, *n*(%)	1 (6.7)
Fever, *n*(%)^#^	22 (91.7)
Abdominal pain, *n*(%)^#^	23 (95.8)
Arthralgia/arthritis, *n*(%)^#^	14 (58.3)
Myalgia, *n*(%)^#^	0 (0.0)
Thoracic pain, *n*(%)^#^	2 (8.3)
Testicular involvement, *n*(%)^#^	0 (0.0)
**IgA vasculitis**	
Age at diagnosis/onset, mean ± SD (years)^$^	10.5 ± 4.1
Onset of IgA vasculitis before FMF, n(%)	4 (8.7)
Associated inflammatory disease, n(%)	
PAN	1 (2.2)
Protracted febrile myalgia	1 (2.2)
Purpura, n(%)	46 (100.0)
Abdominal pain, n(%)	33 (71.7)
Intussusception, n(%)	4 (8.7)
Arthralgia/arthritis, n(%)	33 (71.7)
Renal involvement, n(%)	24 (52.2)
Fever, n(%)	19 (41.3)
Central nervous system involvement, n(%)	2 (4.3)
Splenomegaly, n(%)	7 (15.2)
Myalgia, n(%)	5 (10.9)
Histology showing vasculitis, n(%)^*^	15 (50.0)
Histology showing IgA deposits, n(%)^*^	7 (23.3)
**Treatment**	
Corticosteroids, n(%)^*^	16 (53.3)
Anti IL1, n(%)^*^	0 (0.0)
Cyclophosphamide, n(%)^*^	1 (3.3)
Plasmapheresis, n(%)^*^	2 (6.7)
Symptomatic, n(%)^*^	14 (46.7)

$ *Data available for 41 patients, @ 28 patients*,

** *15 patients*,

# *24 patients*,

* *30 patients*.

**Table 3:** Main clinical characteristics at diagnosis of the 46 patients with IgA vasculitis and FMF, as compared to the patients with IgA vasculitis alone.

**Characteristics**	**Review of the literature IgA vasculitis + FMF, *n* = 46**	**IgA vasculitis without FMF, *n* = 254, Peru et al**.	**IgA vasculitis without FMF, *n* = 150, Trapani et al**.	**IgA vasculitis without FMF, *n* = 78, Calvino et al**.	**IgA vasculitis without FMF, *n* = 100, Saulsbury et al**.	***p*-value ^#^**
**Number of men/women (ratio) ^$^**	21/20 (1.1)	147/107(1.4)	95/55(1.7)	36/42 (0.86)	57/43 (1.33)	0.4278
**IgA vasculitis**						
Age at diagnosis/onset, mean ± SD (years) ^$^	10.5 ± 4.1	8.7 ± 3.6	6.1 ± 2.7	6.2 ± 3.1	5.9 ± 2.9	–
Purpura, *n*(%)	46 (100.0)	254 (100.0)	150 (100.0)	78 (100.0)	100 (100.0)	1
Abdominal pain, *n*(%)	33 (71.7)	144 (56.7)	77 (51.3)	57 (73.1)	63 (63.0)	0.0803
Intussusception, *n*(%)	4 (8.7)	8 (3.1)	1 (0.7)	1 (1.3)	0 (0.0)	0.0147
Arthralgia/arthritis, *n*(%)	33 (71.7)	168 (66.1)	111 (74.0)	61 (78.2)	82 (82.0)	0.9105
Renal involvement, *n*(%)	24 (52.2)	76 (29.9)	81 (54.0)	42 (53.8)	40 (40.0)	0.1415
Central nervous system involvement, *n*(%)	2 (4.3)	NA	4 (2.7)	NA	3 (3.0)	0.6343
Histology showing vasculitis, *n*(%)^*^	15 (50.0)	NA	NA	NA	NA	–
Histology showing IgA deposits, *n*(%)^*^	7 (23.3)	NA	NA	4 (5.1)	NA	–
**Treatment**						
Corticosteroids, *n*(%)^*^	16 (53.3)	86 (33.9)	19 (12.7)	18 (23.1)	57 (57.0)	0.5870
Cyclophosphamide, *n*(%)^*^	1 (3.3)	12 (4.7)	NA	1 (1.3)	NA	1

# *p-value was calculated by comparing patients from the review of the literature to the total number of patients described by Peru et al., Trapani et al., Calvino et al., and Saulsbury et al*.

$ *Data available for 41 patients in our review of the literature*.

* *Data available for 30 patients in our review of the literature*.

Simultaneous occurrence of IgA vasculitis and FMF was described in 46 patients in 12 retrospective studies from the literature (3–5, 14, 20–28) (Table 2). An additional 38 patients were reported, but case descriptions were unavailable (18, 29–35). Only two studies defined IgA vasculitis according to the 1990 ACR criteria (5, 27) and no study defined IgA vasculitis according to the 2010 EULAR criteria. In our analysis, we defined renal involvement according to the 2010 EULAR criteria.

Clinical features of FMF are detailed in Table 2. Almost all patients were of Turkish or Jewish descent. The mean age at diagnosis was 6.8 years for FMF and 10.5 years for IgA vasculitis. The men to women ratio was 1.1. Among the 15 patients whose *MEFV* status was reported, 80% had at least one p.Met694Val mutation, and 46.6% were homozygous for p.Met694Val. All patients with FMF and IgA vasculitis developed purpura. In addition, 71.7% had abdominal pain, 8.7% had an intussusception, 71.7% had joint involvement, and 52.2% had renal involvement. Corticosteroids were used in 53.3% of patients. Other treatments were rarely prescribed. A 7-year-old patient received plasmapheresis and corticosteroids for severe IgA vasculitis with renal involvement, intussusception, and respiratory failure requiring mechanical ventilation; the latter was due to atelectasis, pleural effusion, and subpleural nodules caused by infection or vasculitis (24). Another 10-year-old patient received cyclophosphamide and corticosteroids for cerebral vasculitis (22). Finally, a 7-year-old girl received prednisone, azathioprine, colchicine, and plasmapheresis for relapsing IgA vasculitis with renal involvement and severe abdominal pain (28).

Histological analysis was available for 30 patients. Vasculitis was identified in 50% of biopsies (skin *n* = 15 with leukocytoclastic vasculitis in nine, gastro-intestinal tract *n* = 1, muscle *n* = 1). IgA deposits were identified in 23.3% of biopsies (skin *n* = 3, kidney *n* = 4).

Of interest, FMF symptoms were absent in four patients before the onset of IgA vasculitis. In these patients, FMF was diagnosed at the same time or up to 1 year after IgA vasculitis (20, 22, 24, 26). One of them had central nervous system (CNS) involvement (22), and two others developed one (24) and three (26) episodes of intussusception.

Description of FMF was available for 24 out of 46 patients. It did not differ from previous reports. We also compared these 24 patients with previous cohorts of patients with IgA vasculitis (Table 3) (36–39). The mean age at diagnosis (10.5 years) was higher in patients with both FMF and IgA vasculitis as compared to patients with IgA vasculitis (mean age varying between 5.9 and 8.7 years). The men to women ratio was not different (*p* = 0.43). The main clinical characteristics and rate of corticosteroid use in IgA vasculitis were similar between both groups (*p* > 0.05). The only statistical difference pertained to the increased prevalence of intussusception in FMF patients (8.7%), compared to intussusception rates reported in isolated IgA vasculitis (0 to 3%) (Table 3).

### FMF and Polyarteritis Nodosa (PAN) (Tables 4, 5)

The prevalence of PAN varies between studies, from 4.7 to 31/1 000 000 depending on the study population and diagnostic criteria (16, 40). The prevalence of PAN among FMF patients was assessed in three studies and ranged from 0.9 to 1.4% (4, 5, 19, 41). Only Tekin et al. compared the prevalence of PAN between FMF patients and the general population. In two Turkish hospitals, PAN was diagnosed in 0.004% patients without FMF compared to 1.3% patients with FMF (5).

**Table 4:** Main clinical characteristics at diagnosis of the 61 patients with PAN and FMF.

**Characteristics**	**Review of the literature, PAN + FMF, *n* = 61**
**Number of men/women (ratio)**	47/13 (3.6)
**Ethnicity**, ***n*****(%)**	
Turkish	43 (70.5)
Non-ashkenazi jews	12 (19.7)
Armenian	2 (3.3)
Other	4 (6.5)
**FMF**	
Family history of FMF, *n*(%)	12 (19.7)
Age at diagnosis/onset, mean ± SD (years)^@^	8.1 ± 4.3
Genotyping of MEFV^**^	
**Two pathogenic variants**	17 (73.9)
M694V/ M694V, *n*(%)	12 (52.2)
M694V/V726A, *n*(%)	2 (8.7)
M694V/M680I, *n*(%)	3 (13.0)
**One pathogenic variant**	4 (17.4)
M694V/-, *n*(%)	4 (17.4)
**Variants of unknown pathogenicity**	1 (4.3)
E148Q/E148Q, *n*(%)	1 (4.3)
**Normal**	1 (4.3)
Fever, *n*(%)^#^	28 (90.3)
Abdominal pain, *n*(%)^#^	29 (93.5)
Arthralgia/arthritis, *n*(%)^#^	25 (80.6)
Thoracic pain, *n*(%)^#^	2 (6.5)
**PAN**	
Age at diagnosis/onset, mean ± SD (years)	17.9 ± 8.5
Concomitant vasculitis, *n*(%)	
IgA vasculitis	1 (1.6)
Behçet's disease	1 (1.6)
**Hepatitis B infection**, ***n*****(%)**	4 (6.6)
Weight loss ≥4 kg, *n*(%)	13 (21.3)
**Fever**, ***n*****(%)**	31 (50.8)
**Hypertension**, ***n*****(%)**	30 (49.2)
**Cutaneous involvement (livedo, purpura, subcutaneous nodules, erysipelas-like erythema)**, ***n*****(%)**	26 (42.6)
Central nervous system involvement, *n*(%)	19 (31.1)
Peripheral neuropathy, *n*(%)	11 (18.0)
**Arthralgia/arthritis**, ***n*****(%)**	12 (19.7)
**Myalgia**, ***n*****(%)**	45 (73.8)
Abdominal pain, *n*(%)	31 (50.8)
Hepatomegaly, *n*(%)	5 (8.2)
Splenomegaly, *n*(%)	6 (9.8)
Gastro-intestinal bleeding, *n*(%)	5 (8.2)
Testicular pain, *n*(%)	1 (1.6)
Cardiac involvement, *n*(%)	4 (6.6)
**Renal involvement**	30 (49.2)
**Suspected glomerular involvement**, ***n*****(%)**	21 (34.4)
**Perirenal hematoma**, ***n*****(%)**	30 (49.2)
Abnormal renal arteriography, *n*(%)	35 (57.4)
Histology compatible with PAN, *n*(%)	39 (63.9)
**Treatment ^$^**	
Embolization, *n*(%)	3 (5.9)
**Corticosteroids**, ***n*****(%)**	49 (96.0)
Cyclophosphamide, *n*(%)	31 (60.8)
Azathioprine, *n*(%)	10 (19.6)
Non-steroidal anti-inflammatory drugs /aspirin, *n*(%)	3 (5.9)
Methotrexate, *n*(%)	1 (2.0)
Intravenous Immunoglobulins, *n*(%)	1 (2.0)
Interferon, *n*(%)	1 (2.0)
**Outcome**	
Remission, *n*(%)	55 (90.2)
Death due to vasculitis, *n*(%)	6 (9.8)

# *Data for 31 patients with FMF and PAN, @ 41 patients*,

** *23 patients*,

$ *51 patients*.

**Table 5:** Main clinical characteristics of PAN in the 61 patients with PAN and FMF, as compared to patients with idiopathic PAN described by Pagnoux et al.

**Characteristics of PAN**	**Review of the literature, PAN + FMF, *n* = 61**	**Idiopathic PAN, *n* = 225**	***p*-value**
Number of men/women (ratio)	47/13 (3.6)	137/88 (1.6)	0.012
Age at diagnosis/onset, mean ± SD (years)	17.9 ± 8.5	50.9 ± 17.8	–
Hepatitis B infection, *n*(%)	4 (6.6)	0 (0)	–
Weight loss ≥4 kg, *n*(%)	13 (21.3)	149 (66.2)	<0.001
Fever, *n*(%)	31 (50.8)	136 (60.4)	0.176
Hypertension, *n*(%)	30 (49.2)	–	–
Cutaneous involvement (livedo, purpura, subcutaneous nodules, erysipelas-like erythema), *n*(%)	26 (42.6)	130 (57.8)	0.035
Central nervous system involvement, *n*(%)	19 (31.1)	11 (4.9)	<0.001
Peripheral neuropathy, *n*(%)	11 (18.0)	153 (68.0)	<0.001
Arthralgia/arthritis, *n*(%)	12 (19.7)	106 (47.1)	<0.001
Myalgia, *n*(%)	45 (73.8)	139 (61.8)	0.083
Abdominal pain, *n*(%)	31 (50.8)	62 (27.6)	<0.001
Hepatomegaly, *n*(%)	5 (8.2)	–	–
Splenomegaly, *n*(%)	6 (9.8)	–	–
Testicular pain, *n*(%)	1 (1.6)	18 (13.1)	0.087
Cardiac involvement, *n*(%)	4 (6.6)	46 (20.4)	0.012
Renal involvement, *n*(%)	30 (49.2)	Renal spared mostly	–
Suspected glomerular involvement, *n*(%)	21 (34.4)	–	–
Perirenal hematoma, *n*(%)	30 (49.2)	–	–
Abnormal renal arteriography, *n*(%)^#^	35 (57.4)	59 (62.8)	0.502
Histology compatible with PAN, *n*(%)^*^	39 (63.9)	–(70.1)	0.347

# Data were available for only 94 patients with idiopathic PAN

* *The percentage reported in the publication by Pagnoux et al. did not discriminate between idiopathic and HBV-related PAN*.

Simultaneous occurrence of FMF and PAN was described in a total of 61 patients from 25 retrospective studies (Table 4) (4, 5, 15, 21, 27, 41–60). Seven other patients were described, but the corresponding full texts were unavailable (61–63). Most papers did not specify the criteria used for the diagnosis of PAN. However, 20 patients (32.8%) developed glomerular involvement in disfavor of PAN. Furthermore, seven patients (11.5%) did not fulfill the 1990 ACR criteria for PAN.

Of note, PAN was related to hepatitis B infection in 6.6% of patients. Clinical features of FMF are detailed in Table 4. Of the 61 patients, 90.2% were Turkish or Jewish. The mean age at diagnosis was 8.1 years for FMF and 17.9 years for PAN. The men to women ratio was 3.6. Among the 23 patients whose *MEFV* status was reported, 91.3% were at least heterozygous for p.Met694Val, whereas 52.2% were homozygous for the latter.

In terms of clinical manifestations, 6.6% of patients suffered from cardiac involvement reported as “carditis.” Half of patients described abdominal pain, with gastro-intestinal bleeding in five patients (8.2%): four with blood in the stool, one with bloody diarrhea. One third of patients presented central nervous system involvement, compared to 18% with peripheral nervous system involvement. Renal involvement occurred in 49.2% of patients, as defined by the presence of hematuria and/or proteinuria and/or increase in creatinine. The creatinine value was missing in many reports, preventing further evaluation. A perirenal hematoma developed in 49.2% of patients. Glomerular involvement affected 20 (32.8%) patients, suggesting an alternative diagnosis such as microscopic polyangiitis: eight patients had biopsy-proven glomerulonephritis, four had a nephrotic syndrome with no renal biopsy, two had “marked hematuria and proteinuria” but refused biopsy, two had red blood cell casts without proteinuria, two had hematuria with proteinuria, and two had hematuria without proteinuria or casts. Antineutrophil cytoplasmic antibodies were tested in only five patients and were negative (41). Abnormalities on renal arteriography, such as aneurysm or vascular occlusion, were reported in 57.4% of patients.

Histological analysis was compatible with PAN in 63.9% of cases. Compatible histology was defined as the presence of leucocytes in small or medium-sized arteries according to the 1990 ACR criteria for PAN. Alternatively, the following statements were accepted: “histology compatible with PAN,” “leukocytoclastic vasculitis,” or “necrotizing vasculitis.” Corticosteroids were used in 96% of patients, cyclophosphamide in 60.8%, and azathioprine in 19.6%. Remission was achieved in 90.2% of patients. Conversely, 9.8% of patients died due to PAN.

In order to identify distinctive features of FMF-associated PAN, in which concurrent hepatitis B (HBV) infection was identified in a minority (6.6%), patients with both FMF, and PAN were compared to 225 patients with non-HBV-related PAN reported by Pagnoux et al. (64) (Table 5). PAN in FMF patients was generally diagnosed during the second or third decade of life (mean age 17.9 years), as compared to idiopathic PAN which was diagnosed during the fourth or fifth decade (mean age 50.0 years). The men to women ratio was higher in FMF-associated PAN (3.6 vs. 1.6 in idiopathic PAN, *p* < 0.05). Patients with both PAN and FMF presented statistically more central nervous system involvement and abdominal pain, but less peripheral neuropathy, weight loss, arthralgia, and cardiac involvement (*p* < 0.05). Renal involvement was also more frequent, with perirenal hematoma in half of patients. Similar results were obtained after exclusion of patients with glomerular involvement, with abdominal pain in 71% of patients (*p* < 0.001), perirenal hematomas in 50%, and CNS involvement in 25% (*p* = 0.001), and less peripheral neuropathy (14%, *p* < 0.001) and weight loss (36%, *p* = 0.001) compared to idiopathic PAN. However, the incidence of arthralgia did not differ significantly between FMF-associated and idiopathic PAN (*p* = 0.13).

Some reports suggested a more favorable prognosis in patients with both FMF and PAN compared to patients with PAN alone (7). Pagnoux et al. reported a 92% survival rate at 1 year in patients with idiopathic PAN, which decreased to 74% at 10 years (64). In comparison, patients with both FMF and PAN had a 90% remission rate, but follow-up rarely exceeded 1 year. Given this limitation, we cannot conclude that the prognosis of FMF-associated PAN differs from idiopathic PAN.

### FMF and Behçet's Disease (Table 6)

Depending on the country, the region, the method of investigation, and the classification criteria, the prevalence of Behçet's disease varies widely between studies, from 0.27 to 420/100 000 inhabitants (65). The highest prevalence is reported in Turkey and Israel, with 80 to 420/100 000 inhabitants and 15.2 to 120/100 000 inhabitants, respectively (65).

**Table 6:** Main clinical characteristics at diagnosis of the 46 patients with BD and FMF.

**Characteristics**	**Review of the literature, BD + FMF, *n* = 46**
**Number of men/women (ratio) ^#^**	22/22 (1)
**Ethnicity**, *n*(**%)**	
Non-Ashkenazi Jews	18 (39.2)
Iraqi or Turkish Jews	14 (30.4)
Turkish	2 (4.3)
Palestinian Arab	2 (4.3)
Iranian	1 (2.2)
Japanese	1 (2.2)
Other	8 (17.4)
**FMF**	
Family history of FMF, *n*(%) ^$^	31 (75.6)
Age at diagnosis/onset, mean ± SD (years) &	12.8 ± 2.6
Genotyping of *MEFV* ^**^	
**Two pathogenic variants**	2 (33.3)
M694V/M694V, *n*(%)	1 (16.7)
V726A/V726A, *n*(%)	1 (16.7)
**One pathogenic variant**	2 (33.3)
E148Q/M694I, *n*(%)	1 (16.7)
M694V/-, *n*(%)	1 (16.7)
**Variants of unknown pathogenicity**	2 (33.3)
E148Q/P369S, *n*(%)	1 (16.7)
E148Q/E148Q, *n*(%)	1 (16.7)
Fever, *n*(%)€	24 (53.3)
Abdominal pain, *n*(%)€	33 (73.3)
Arthralgia/arthritis, *n*(%)€	35 (77.8)
Myalgia, *n*(%)€	13 (28.9)
Thoracic pain, *n*(%)€	22 (48.9)
Testicular pain, *n*(%)€	3 (6.7)
Erysipelas-like erythema, *n*(%)€	6 (13.3)
**BD**	
Age at diagnosis/onset, mean ± SD (years)^#^	21.3 ± 4.0
Disease form, *n*(%)	
Complete	22 (48.0)
Incomplete	24 (52.0)
Concomitant inflammatory disease, *n*(%)	
PAN	1 (2.2)
Amyloidosis	1 (2.2)
HLA-B51 positivity, *n*(%) @	–(51.7)
BD preceding FMF, *n*(%)^#^	2 (4.5)
Genital ulcers, *n*(%)^#^	22 (50.0)
Cutaneous manifestations, *n*(%)^#^	38 (86.4)
Positive pathergy test, *n*(%)^#^	8 (18.2)
Ophthalmologic manifestations, *n*(%)^#^	28 (63.6)
Renal involvement, *n*(%)^#^	6 (13.6)
CNS involvement, *n*(%)^#^	18 (40.9)
Arthralgia/arthritis, *n*(%)^#^	19 (43.2)
**Treatment ^#^**	
Colchicine	39 (88.6)
Corticosteroids	7 (15.9)
Non-steroidal anti-inflammatory drugs	1 (2.3)
Cyclophosphamide	1 (2.3)
Azathioprine	1 (2.3)
Cytotoxic drugs	2 (4.5)
Methotrexate	1 (2.3)
Sulfasalazine	1 (2.3)
Anti-IL1	1 (2.3)

*€Data for 45 patients*,

# *44 patients, and 43 patients*,

$ *41 patients, @ four patients*,

** *six patients*.

The prevalence of BD in patients with FMF was investigated in three studies. In Turkey, Tunca et al. reported a prevalence of 0.5% among 2,838 FMF patients, which equates to ~420/100 000 inhabitants as previously reported in the general Turkish population (19, 65). In Israel, two studies described a prevalence of 0.4 to 0.56% in FMF patients (20, 66), which exceeded BD prevalence in the general Israeli population (65). However, these studies did not use appropriate controls from the same Israeli centers, such that the prevalence reported could be due to a population effect (67, 68).

In 2017, Watad et al. noted a significantly increased prevalence of FMF in patients with BD (5.83 vs. 0.23% in the control group) (69). In patients with BD, FMF was associated with female sex, as supported by a men to women ratio of 0.4 (OR 177 for females vs. 8.4 for males) compared to 1.1 in BD in general. BD was also an independent risk factor for FMF in multivariate analysis (OR 25).

Simultaneous occurrence of FMF and BD was described in a total of 46 patients from seven studies. Thirty-nine patients were reported by Schwartz et al. (Table 6) (6, 48, 66, 70–73). Of these 46 patients, 23 had an incomplete form of BD, but were not specifically identified in the analysis by Schwartz et al. In all studies, the diagnosis of complete BD was based on the ISG criteria. Clinical characteristics of FMF associated with BD did not differ from FMF in general. Only six patients were genotyped for *MEFV*. The mean age at diagnosis was 12.8 years for FMF and 21.3 years for BD. The men to women ratio was 1. Birlik et al. reported a Turkish patient with complete BD and HLA-B27-negative sacroiliitis, treated with colchicine 1.5 mg daily and sulfasalazine (70). Korkmaz et al. reported an American patient with complete BD and PAN treated with colchicine 2 mg daily and cyclophosphamide (48). Bilginer et al. reported a Turkish patient with FMF and complete BD complicated by AA amyloidosis, who was successfully treated with anakinra (71). Matsuda et al. described a Japanese patient with both FMF and incomplete BD effectively treated with colchicine 0.5 mg daily (72). Mobini et al. described a patient with FMF and BD who responded favorably to colchicine and azathioprine (73). Ben Chetrit et al. presented two patients with FMF and BD, although BD characteristics were not detailed (66).

Schwartz et al. compared 39 patients with FMF and complete or incomplete BD with 29 BD controls and 100 FMF controls (6). They described more Iraqi and Turkish Jews (*p* < 0.01), less abdominal attacks (*p* < 0.02), a similar severity of FMF, a lower response to treatment (*p* < 0.05) but also a lower adherence to treatment (*p* < 0.05) in patients with FMF and BD, as compared to 100 FMF controls. Furthermore, there were more North African Jews, more frequent family history of BD, and more cutaneous, gastro-intestinal and CNS involvement in patients with FMF and BD as compared to 29 BD controls (*p* < 0.02), with similar disease severity and treatments.

### Other Vasculitis (Tables 7, 8)

Vasculitis other than IgA vasculitis, PAN, and BD were rarely reported in patients with FMF. Cogan syndrome was described in one patient (patient 10) (83). Large vessel vasculitis, namely Takayasu arteritis, was described in two patients (patient eight and nine). Patient eight relapsed three times, whereas patient nine had prolonged remission (81, 82).

**Table 7:** Main features of FMF in 14 patients with a vasculitis other than IgA vasculitis, PAN, and BD.

**Patient**	**Sex**	**Age at inclusion (years)**	**References**	**Ethnicity**	**Family history**	***MEFV* mutation**	**Age at onset of FMF symptoms (years)**	**Age at diagnosis of FMF (years)**	Clinical manifestations of FMF^*^	**Diagnosis of FMF before vasculitis**	**Colchicine before vasculitis onset**	**Age at initiation of Colchicine (years)**	**Mean daily dose of Colchicine (mg)**	**Inactive FMF before vasculitis onset**
1	M	28	Schlesinger et al. (74)	NAJ	FMF: brother	-	18	18	FEV, ABD, ART, PLE	Yes	Yes	24	Stopped at 27	Yes
2	W	29	Serrano et al. (75)	Spanish	FMF and AA amyloidosis: sister	-	2	19	FEV, ARI, CUT	Yes	Yes	19	-	Yes
3	W	8	Oguzkurt et al. (76)	Turkish	Consanguinity	-	7	7	FEV, ART, ABD	Yes	Yes	7	-	Yes
4	W	40	Braun et al. (77)	Jewish	-	-	childhood	childhood	-	Yes	Yes	Childhood	2, 5	Yes
5	W	53	Cefle et al. (78)	Turkish	FMF: sister and brother	-	20	46	FEV, ABD, PLE	Yes	Yes	46	Irregular intake	-
6	M	53	Cocco (79)	Spanish	Probable FMF: father	M694V/E148Q	53	53	-	Concomitant	No	-	-	-
7	M	39	Satoh et al. (80) (abstract)	Japanese	-	-	childhood	38	FEV, ABD, THO	Yes	Yes	38	-	-
8	M	28	Zihni et al. (81)	Turkish	FMF: sister	M694V/V726A	9	9	FEV, ABD	Yes	Yes	9	1, 5	Yes
9	M	24	Alibaz-Oner et al. (82)	Turkish	-	R314R/E474E/ Q476Q/D510D	-	17	FEV, THO	Yes	Yes	17	1, 5	Yes
10	M	-	Zenone et al. (83)	-	-	-	-	-	-	-	-	-	-	-
11	M	-	Luger et al. (84)	-	-	-	-	-	-	-	-	-	-	-
12	M	31	Komatsu et al. (85)	Japanese	-	G2082A/-	16	31	FEV, ABD, MUS, CUT	Concomitant	No	-	-	-
13	M	41	Tekin et al. (86)	Turkish	-	M694V/M694V	-	-	-	-	-	-	-	-
14	W	32	Ozates et al. (87)	Turkish	Yes, not detailed	M694V/M694V	-	31	-	Yes (2 months)	Yes	31	2	-

* *FEV, fever; ABD, abdominal pain; ART, arthralgia; ARI, arthritis; CUT, skin rash; THO, thoracic pain; TES, testicular pain/orchitis; ERY, erysipelas-like erythema; MUS, myalgia; PLE, pleural pain; NAJ, Non-Ashkenazi Jews*.

**Table 8:** Main features and evolution of vasculitis other than IgA vasculitis, PAN, and BD in 14 patients with FMF.

**Patient**	**Vasculitis**	**Age at diagnosis of vasculitis (years)**	**Manifestations of vasculitis**	**Ischemic or hemorrhagic events**	**Histology**	**Treatment of vasculitis**	**Efficacy of treatment**	**Age at last follow-up (years)**
1	Medium arteries	28	Severe muscle pain with normal CK, macro hematuria, prot 3 g/24 h, elevated CRP, negative throat culture	–	Muscle and normal skin: arteritis of medium vessels, fibrinoid necrosis; kidney: proliferative diffuse glomerulonephritis with deposits of immune complexes, C3, IgM	Non-steroidal anti-inflammatory drugs, colchicine	Remission	29
2	Medium arteries	29	Occlusion of anterior and right proximal coronary arteries	Acute myocardial infarction (death)	Heart: amyloid deposits in intramyocardial arteries; vasculitis of large epicardial arteries, acute inflammation, adventitial palisading granulomas	Angioplasty	Death	29
3	Medium arteries	8	Fever, hepatomegaly, thick and large gallbladder, intra-abdominal lymphadenopathy, elevated CRP	–	Liver and gallbladder: focal bridging and nodular transformation, arteritis of medium vessels, fibrinoid necrosis, obliteration; lymph node: follicular hyperplasia	CT	Remission	0.5
4	Small vessels?	40	Abdominal pain, ascites, pleuritis, electromyography-proven neuropathy, purpura	Intra alveolar hemorrhage (ICU)	Skin: acute leukocytoclastic vasculitis	CT, CYC	Remission	40
5	Small vessels? (Pauci-immune GN like) + amyloidosis	51	Macrohematuria, prot 6 g/24 h, elevated CRP, negative ANCA	–	Kidney: amyloidosis, necrotizing crescentic glomerulonephritis, granulomatous vasculitis, no Ig or complement deposit	Colchicine 2 mg/day, AZA	Remission	53
6	Small vessels (MPA-like)	53	Fever, purpura, abdominal pain, myalgia, arthritis, severe Raynaud syndrome, LBBB, elevated CRP, positive anti-myeloperoxidase antibodies, mild hepatosplenomegaly, and pleural, and pericardial effusion	–	Skin: vasculitis of small vessels, necrosis	Colchicine 1 mg/day	Partial remission (persistence of myalgia and arthralgia)	54
7	Small vessels	39	Frosted branch angiitis with retinal vein occlusion	Retinal hemorrhage	–	CT, antiviral and antibacterial agents	Remission	3.5
8	Large vessels (Takayasu)	22	Symptomatic stenosis of the proximal left common carotid artery and subclavian artery, stenosis of the right common carotid artery and celiac artery (angiography); ascending and descending thoracic and abdominal aortitis; skin; (3 relapses)	–	Skin: vasculitis	CT, MTX, CYC, AZA, INF	Remission	28
9	Large vessels (Takayasu)	24	Asymptomatic high-grade stenosis of the left common carotid artery and subclavian artery, total occlusion of the right axillary artery (angiography)	–	–	CT, AZA, CYC	Remission	24.5
10	Cogan syndrome	–	–	–	–	–	–	–
11	–	–	Brain infarction during a typical FMF attack	Brain stem infarction	–	–	–	–
12	Small arteries	31	Subcutaneous nodules with every FMF attack since he was 27 years old; presence of a T61I/- mutation in *TNFRSF1A*	–	Skin: necrotizing vasculitis of small arteries, obliteration	Colchicine 0.75 mg/day	Remission	31
13	Small vessels? + amyloidosis	41	Purpura, elevated CRP, prot 0.7 g/24 h	–	Skin: leukocytoclastic vasculitis, kidney: amyloidosis	CT	–	41
14	Small vessels	32	Frosted branch angiitis (1 relapse)	Retinal and sub hyaloid hemorrhage	–	CT, hyaloidotomy	Remission	33

*ANCA, anti-neutrophil cytoplasmic antibodies; AZA, azathioprine; CRP, C-reactive Protein; CT, corticosteroids; CYC, cyclophosphamide; GN, glomerulonephritis; ICU, intensive care unit; Ig, immunoglobulins; INF, infliximab; LBBB, left bundle branch block; MTX, methotrexate; MPA, microscopic polyangiitis; PAN, polyarteritis nodosa; PLA, hydroxychloroquine; prot, proteinuria; HBV, hepatitis B virus*.

Unclassified vasculitis was described in a total of 11 FMF patients from various case reports (74–80, 84–87). FMF was associated with unclassified medium vessel vasculitis in three patients (patient 1, 2, 3). Schlesinger et al. described a patient with cutaneous vasculitis and diffuse proliferative glomerulonephritis suggesting post-streptococcal vasculitis. However, the presence of severe myalgia, the absence of previous streptococci infection, and the absence of IgG deposits in the kidney was more consistent with FMF-related vasculitis (74). Serrano et al. described a patient diagnosed simultaneously with FMF and AA amyloidosis at 19 years old (75). Although her FMF symptoms resolved on colchicine, progressive renal failure due to AA amyloidosis led to renal transplantation at 24 years old. She was subsequently treated with cyclosporine, mycophenolate mofetil and corticosteroids. Five years later, she developed coronary artery vasculitis. Although cyclosporine can cause small vessel vasculitis, an iatrogenic cause was deemed less likely as the patient had a medium vessel vasculitis that was more probably FMF-related (75). Finally, Oguzkurt et al. described a patient with HBV-negative hepatobiliary pseudo-PAN with hepatic nodular transformation (76).

The co-occurrence of FMF and unclassified small vessel vasculitis was described in seven patients (patients 4, 5, 6, 7, 12, 13, and 14) (77–80, 85–87). Vasculitis phenotypes were heterogeneous. Two patients displayed a frosted branch angiitis (patients seven and 14). Two others were concomitantly diagnosed with vasculitis and amyloidosis (patients five and 13). Patient five was poorly compliant with colchicine, and was eventually diagnosed with renal amyloidosis and granulomatous vasculitis. Patient 13 was a 41-year-old man with ankylosing spondylitis and FMF who presented suddenly with leukocytoclastic vasculitis and renal amyloidosis (78, 86).

Unclassified vasculitis developed during an FMF attack in three patients (patients 6, 11, and 12) (79, 84, 85). Patient 11 had a brain infarction for which investigations, including cerebrospinal fluid analysis, conventional angiography, and leptomeningeal biopsy, confirmed a FMF-related CNS vasculitis (84). Additional information, such as precise description of the stroke and size of affected vessels, was not provided.

## Discussion

IgA vasculitis is the most frequent vasculitis in FMF patients, with a prevalence of 2.7–7%. It was diagnosed later in FMF patients compared to the general population (Table 3). The men to women ratio was unchanged (*p* = 0.43) (34–37). The clinical presentation of both diseases in the same individual was similar to the general population, except for an increased risk of intussusception which affected 8.7% of patients. In agreement with Ben Chetrit et al., we identified leukocytoclastic vasculitis as the predominant histological feature in FMF-associated IgA vasculitis (88).

Although most papers did not specify the criteria used for the diagnosis of IgA vasculitis, all patients fulfilled the 1990 ACR criteria. Indeed, they all had purpura and were <20 years old at disease onset except for one patient. This patient, as described by Balbir et al., displayed abdominal pain and features of leukocytoclastic vasculitis on skin biopsy, thus fulfilling both ACR and EULAR criteria for IgA vasculitis (23). The EULAR criteria were also fulfilled in all patients, except for one with missing clinical data (3). The diagnostic challenge of IgA vasculitis in FMF patients stems from similar age at onset and symptom overlap, namely purpura, abdominal pain, and arthralgia.

In his case series, Ben-Chetrit et al. proposed that purpura in most patients was related to FMF rather than IgA vasculitis (88). In our analysis, we found that most patients had lower extremity purpura typical of IgA vasculitis. All patients but one presented with a single episode diagnosed as IgA vasculitis, which contrasted with their usual FMF symptoms. Among the 30 patients with histological data available, 19 (63%) had vasculitis (*n* = 15) and/or IgA deposits (*n* = 7) and/or renal histology showing glomerulonephritis/IgA vasculitis nephritis (*n* = 6). It is striking that IgA deposits were identified in only 23.3% of histological samples, while they were previously described in 66.4% of patients with IgA vasculitis (11). However, this feature is not very sensitive. Furthermore, histological data in FMF-associated IgA vasculitis are scarce. As a result, the absence of IgA deposits does not exclude a diagnosis of IgA vasculitis. Moreover, half of patients with FMF and IgA vasculitis required treatment with corticosteroids, and 8.7% developed an intussusception. We therefore recommend that clinicians who suspect IgA vasculitis in FMF patients should not rule out the diagnosis based on absent IgA deposits on skin biopsy. If the clinical presentation is suggestive of IgA vasculitis, they should manage the patient accordingly given the increased risk of digestive complications in FMF-associated IgA vasculitis.

Finally, among the 46 patients with FMF and IgA vasculitis, four patients (9%) developed FMF symptoms concurrent to or after the onset of IgA vasculitis, whereas nine patients (20%) were retrospectively diagnosed with FMF when they presented with IgA vasculitis (4). This suggests that patients with IgA vasculitis in areas with high prevalence of FMF should be questioned about current and past symptoms suggestive of FMF. In the 15 patients with FMF-associated IgA vasculitis and *MEFV* sequencing, 75% carried two pathogenic mutations. Overall, our data suggest that FMF, especially in the presence of two pathogenic mutations, might predispose to IgA vasculitis, or lead to the development of a vasculitis resembling IgA vasculitis. Whether this vasculitis presents less frequently with IgA deposits on biopsy in FMF patients remains unknown, due to the paucity of histological data available.

PAN is the second most frequent vasculitis associated with FMF, affecting 0.9–1.4% of patients. It is not related to hepatitis B infection, which was documented in only 6.6% of patients. Moreover, FMF-associated PAN presents striking differences with idiopathic PAN, namely younger age at onset, an increased men to women ratio, and a higher rate of perirenal hematoma and central nervous system involvement. It is noteworthy that almost half of patients displayed glomerular involvement or did not fulfill the 1990 ACR criteria for PAN, suggesting an alternative diagnosis. Although patients with FMF and PAN presented more frequently with abdominal pain concomitant to other PAN symptoms, the latter could be due either to vasculitis or FMF, which limits its interpretation.

Medium vessel vasculitis due to deficiency in adenosine deaminase 2 (DADA2) and FMF-associated PAN both have an increased risk of central nervous system involvement compared to idiopathic PAN. We could therefore hypothesize that some patients with FMF and a PAN-like vasculitis may actually have underlying DADA2 (89, 90). However, these patients had increased risk of perirenal hematoma at the forefront, with no family history of vasculitis, while patients with DADA2-related vasculitis experience more ischemic cerebrovascular accidents with only rare bleeding complications, and no perirenal hematoma reported so far (89, 90).

The aforementioned findings were not influenced by the presence of pathogenic *MEFV* mutations. Furthermore, FMF-associated PAN presents such distinctive clinical features that it could represent a unique medium vessel vasculitis specific to FMF. As a result, it seems more appropriate to refer to patients with both PAN and FMF as having a PAN-like medium vessel vasculitis rather that classical PAN, especially since half of them cannot be classified as PAN based on glomerular involvement and/or incomplete ACR criteria. Future studies with long-term follow-up are needed to better define the prognosis of these patients.

After IgA vasculitis and PAN, BD may be the third most prevalent vasculitis in FMF patients. In light of the recently conceptualized continuum between autoimmune and autoinflammatory diseases, BD is considered to be a combination of both (91). The hypothesis of a common pathophysiological pathway between BD and FMF is supported by the efficacy of colchicine in both diseases. Moreover, many studies from Mediterranean countries using different methodologies reported a variable but overall increased prevalence of one *MEFV* mutation in non-FMF patients with BD; heterozygous or homozygous status for pathogenic MEFV mutations could therefore predispose to the development of BD. However, our literature analysis cannot confirm an association between BD and FMF due to lack of appropriate controls in the studies. Indeed, prevalence of FMF differs not only between Mediterranean countries, but also between regions within a same country, such that every prevalence reported should be compared to the prevalence of BD among comparable patients from the same region.

The men to women ratio in patients with FMF and BD varied between studies. While our analysis found a men to women ratio of one, Watad et al. reported a ratio of 0.4 that was significantly lower than patients with BD alone (69). Patients with BD and FMF presented a mean age at BD onset and diagnosis of 21.3 ± 4.0 years, in accordance with previous reports (92). FMF patients with and without BD presented with a similar FMF phenotype. Conversely, BD in FMF patients had more gastro-intestinal and CNS involvement than BD in general. Symptom overlap between FMF and BD prevents us from drawing conclusions based on the increased rate of gastro-intestinal symptoms. On the other hand, CNS involvement in over two-third of FMF-associated BD is quite interesting. These cases were all reported by Schwartz et al., and involved meningoencephalitis, hemiparesis, vertigo, diplopia, as well as migraine headaches (6).

Very few FMF patients with well-classified vasculitis other than IgA vasculitis, PAN and BD are described in the literature. The co-occurrence of FMF and unclassified vasculitis was reported in 11 patients. In all of them, vasculitis was considered related to FMF. It affected small size vessels (*n* = 7), medium size vessels (*n* = 3), and all size vessels (*n* = 1). *MEFV* gene sequencing was not performed in the majority of cases. Clinical phenotypes were highly variable, with no particular trend. However, five out of 13 patients developed ischemic or bleeding complications. Despite the fact that these cases were considered as FMF-related vasculitis, we believe that there is still insufficient data to exclude a fortuitous association between FMF and unclassified vasculitis.

This systematic review has several limitations. Many papers did not detail the clinical presentation of vasculitis, and thus were excluded. Most cases of vasculitis included for analysis were published before 2000, with scarce histological data. Articles were also very heterogeneous, with some using their own description of organ involvement. The majority did not specify the criteria used for the diagnosis of vasculitis, such that we had to retrospectively evaluate these cases in order to compare them with one another. To avoid any confusion between real FMF and asymptomatic carriers of an MEFV mutation, we excluded reports in which patients did not fulfill the Livneh diagnostic criteria for FMF. Few studies compared the prevalence of FMF-associated vasculitis with comparable subjects from the general population. Concerning BD, only one case series described patients with FMF and BD. Its authors included and analyzed together patients with both complete and incomplete forms of BD, which may have biased the results (6). Moreover, because most cases came from this series, and because the authors used their own definition of organ involvements, no firm conclusions regarding FMF-associated BD can be drawn based on this review. Future studies describing larger cohorts of patients with FMF and vasculitis, with detailed clinical and histological data, are needed.

## Conclusion

In this systematic review, we confirmed that IgA vasculitis is the most prevalent vasculitis in FMF patients with a prevalence of 2.7–7%, followed by PAN with a prevalence of 0.9–1.4%. Unfortunately, we cannot conclude with certainty that BD develops more frequently in FMF patients. A question remains: should we consider these vasculitis as distinct entities in the presence of FMF? We believe that the answer is no for IgA vasculitis, although its onset might be influenced by the presence of FMF and *MEFV* mutations. The clinical presentation of FMF and IgA vasculitis is, however, similar to the general population. Identification of IgA deposits on biopsy seems less frequent in FMF patients, but this needs to be confirmed by other studies given the paucity of histological data. Conversely, the answer to the previous question is probably yes for PAN. Indeed, FMF patients with PAN-like vasculitis may actually suffer from a distinct small and medium vessel vasculitis, with younger age at onset and more perirenal hematoma and CNS involvement.

## Author Contributions

SA and SG-L did the first literature search. SA did the detailed literature analysis, the statistical analysis, and wrote the first draft. SA, SG-L, DS, and GG all contributed to the data analysis and interpretation and to drafting and revising the manuscript content. SD-B reviewed the final draft for data interpretation, grammar, and spelling correction.

### Conflict of Interest Statement

None for this specific study. SG-L and GG have received in the past honoraria as occasional consultants for the SOBI and Novartis laboratories (< 5,000 euros). The remaining authors declare that the research was conducted in the absence of any commercial or financial relationships that could be construed as a potential conflict of interest.

## References

[1] Ben-Chetrit E Levy M. Familial mediterranean fever. Lancet. (1998) 351:659–64. 10.1016/S0140-6736(97)09408-7 9500348

[2] de Torre-Minguela C Mesa Del Castillo P Pelegrín P The NLRP3 and pyrin inflammasomes: implications in the pathophysiology of autoinflammatory diseases. Front Immunol. (2017) 8:43 10.3389/fimmu.2017.00043 28191008 PMC5271383

[3] Flatau E Kohn D Schiller D Lurie M Levy E. Schönlein-henoch syndrome in patients with familial mediterranean fever. Arthritis Rheum. (1982) 25:42–7. 10.1002/art.1780250107 7066036

[4] Ozdogan H Arisoy N Kasapçapur O Sever L Calişkan S Tuzuner N. Vasculitis in familial mediterranean fever. J Rheumatol. (1997) 24:323–7. 9034991

[5] Tekin M Yalçinkaya F Tümer N Cakar N Koçak H Ozkaya N. Familial Mediterranean fever–renal involvement by diseases other than amyloid. Nephrol Dial Transplant. (1999) 14:475–9. 10069219 10.1093/ndt/14.2.475

[6] Schwartz T Langevitz P Zemer D Gazit E Pras M Livneh A. Behçet's disease in familial mediterranean fever: characterization of the association between the two diseases. Semin Arthritis Rheum. (2000) 29:286–95. 10.1016/S0049-0172(00)80015-3 10805353

[7] Aksu K Keser G. Coexistence of vasculitides with familial mediterranean fever. Rheumatol Int. (2011) 31:1263–74. 10.1007/s00296-011-1840-z 21547384

[8] Jain A Misra DP Sharma A Wakhlu A Agarwal V Negi VS. Vasculitis and vasculitis-like manifestations in monogenic autoinflammatory syndromes. Rheumatol Int. (2018) 38:13–24. 10.1007/s00296-017-3839-6 29032440

[9] Lightfoot RW Michel BA Bloch DA Hunder GG Zvaifler NJ McShane DJ. The American College of Rheumatology 1990 criteria for the classification of polyarteritis nodosa. Arthritis Rheum. (1990) 33:1088–93. 10.1002/art.1780330805 1975174

[10] Mills JA Michel BA Bloch DA Calabrese LH Hunder GG Arend WP. The American College of Rheumatology 1990 criteria for the classification of Henoch-Schönlein purpura. Arthritis Rheum. (1990) 33:1114–21. 10.1002/art.1780330809 2202310

[11] Ozen S Pistorio A Iusan SM Bakkaloglu A Herlin T Brik R. EULAR/PRINTO/PRES criteria for Henoch-Schönlein purpura, childhood polyarteritis nodosa, childhood Wegener granulomatosis and childhood Takayasu arteritis: ankara 2008. Part II: final classification criteria. Ann Rheum Dis. (2010) 69:798–806. 10.1136/ard.2009.116657 20413568

[12] Criteria for diagnosis of Behçet's disease International study group for behçet's disease. Lancet Lond Engl. (1990) 335:1078–80. 1970380

[13] Livneh A Langevitz P Zemer D Zaks N Kees S Lidar T. Criteria for the diagnosis of familial Mediterranean fever. Arthritis Rheum. (1997) 40:1879–85. 10.1002/art.1780401023 9336425

[14] Cabral M Conde M Brito MJ Almeida H Melo Gomes JA. Protracted febrile myalgia syndrome with henoch-schönlein purpura: an atypical presentation of familial mediterranean fever. Acta Reumatol Port. (2011) 36:69–74. 21483284

[15] Bosacki C Richard O Freycon F Mosnier JF Cathébras P. The association of polyarteritis nodosa and familial Mediterranean fever. Presse Med. (2003) 32:24–6. 12610392

[16] Lane SE Watts R Scott DGI. Epidemiology of systemic vasculitis. Curr Rheumatol Rep. (2005) 7:270–5. 10.1007/s11926-005-0036-5 16045829

[17] Piram M Mahr A. Epidemiology of immunoglobulin A vasculitis (Henoch–Schönlein): current state of knowledge. Curr Opin Rheumatol. (2013) 25:171–8. 10.1097/BOR.0b013e32835d8e2a 23318735

[18] Sohar E Gafni J Pras M Heller H. Familial mediterranean fever. A survey of 470 cases and review of the literature. Am J Med. (1967) 43:227–53. 10.1016/0002-9343(67)90167-2 5340644

[19] Turkish FMF Study Group Familial mediterranean fever (FMF) in Turkey: results of a nationwide multicenter study. Medicine. (2005) 84:1–11. 10.1097/01.md.0000152370.84628.0c 15643295

[20] Caglar MK. Familial Mediterranean fever after recovery from Schönlein-Henoch syndrome. Arthritis Rheum. (1983) 26:1536. 10.1002/art.1780261224 6651905

[21] Lange-Sperandio B Möhring K Gutzler F Mehls O. Variable expression of vasculitis in siblings with familial Mediterranean fever. Pediatr Nephrol. (2004) 19:539–43. 10.1007/s00467-004-1440-1 15015067

[22] Özkaya O Bek K Alaca N Ceyhan M Açikgöz Y Taşdemir HA. Cerebral vasculitis in a child with Henoch–Schönlein purpura and familial Mediterranean fever. Clin Rheumatol. (2007) 26:1729–32. 10.1007/s10067-006-0485-x 17235658

[23] Balbir-Gurman A Nahir AM Braun-Moscovici Y. Vasculitis in siblings with familial Mediterranean fever: a report of three cases and review of the literature. Clin Rheumatol. (2007) 26:1183–5. 10.1007/s10067-006-0323-1 16721494

[24] Unal S Güçer S Kale G Besbas N Özen S Gümrük F. Severe Henoch-Schönlein purpura in a thalassemic patient under deferiprone treatment. Am J Hematol. (2008) 83:165–6. 10.1002/ajh.21052 17712793

[25] Duru NS Civilibal M Karakoyun M Payasli M Elevli M Protracted febrile myalgia in two children with familial Mediterranean fever: protracted febrile myalgia in FMF. Pediatr Int. (2009) 52:e137–40. 10.1111/j.1442-200X.2010.03058.x 20723111

[26] Adiv OE Butbul Y Nutenko I Brik R A typical Henoch-Schonlein purpura: a forerunner of familial Mediterranean fever. Isr Med Assoc J IMAJ. (2011) 13:209–11. 21598807

[27] Girisgen I Sonmez F Koseoglu K Erisen S Yilmaz D. Polyarteritis nodosa and Henoch–Schönlein purpura nephritis in a child with familial mediterranean fever: a case report. Rheumatol Int. (2012) 32:529–33. 10.1007/s00296-010-1766-x 21259007

[28] Nickavar A Ehsanipour F Recurrent Henoch-Schönlein Purpura in familial mediterranean fever. Acta Med Iran. (2008) 46:349–352.

[29] Federgrin A Henoch-Schönlein Syndrome in Children [M.D. thesis]. 2nd ed Hebrew University Medical School, Jerusalem (1961).

[30] Althausen T The false “acute abdomen.” II. Henoch's purpura and abdominal allergy. J Allergy. (1938) 9:209. 10.1097/00000658-193708000-00008 PMC1390498 17857033

[31] Calligaris G. Two familial cases of periodic disease. Minerva Pediatr. (1953) 5:781–4. 13111008

[32] Rotem Y Federgruen A. Schoenlein-Henoch syndrome in familial mediterranean fever. Harefuah. (1962) 62:1–5. 14494360

[33] Eliakim M Rachmilewitz M Rosenmann E Niv A. Renal manifestations in recurrent polyserositis (familial Mediterranean fever). Isr J Med Sci. (1970) 6:228–45. 5506237

[34] Schlesinger M Rubinow A Vardy PA. Henoch-Schönlein purpura and familial mediterranean fever. Isr J Med Sci. (1985) 21:83–5. 3871752

[35] Majeed HA Quabazard Z Hijazi Z Farwana S Harshani F. The cutaneous manifestations in children with familial Mediterranean fever (recurrent hereditary polyserositis). A six-year study. Q J Med. (1990) 75:607–16. 2217666

[36] Peru H Soylemezoglu O Bakkaloglu SA Elmas S Bozkaya D Elmaci AM. Henoch Schonlein purpura in childhood: clinical analysis of 254 cases over a 3-year period. Clin Rheumatol. (2008) 27:1087–92. 10.1007/s10067-008-0868-2 18305976

[37] Trapani S Micheli A Grisolia F Resti M Chiappini E Falcini F. Henoch Schonlein purpura in childhood: epidemiological and clinical analysis of 150 cases over a 5-year period and review of literature. Semin Arthritis Rheum. (2005) 35:143–53. 10.1016/j.semarthrit.2005.08.007 16325655

[38] Calviño MC Llorca J García-Porrúa C Fernández-Iglesias JL Rodriguez-Ledo P González-Gay MA. Henoch-Schönlein purpura in children from northwestern Spain: a 20-year epidemiologic and clinical study. Medicine. (2001) 80:279–90. 10.1097/00005792-200109000-00001 11552081

[39] Saulsbury FT. Henoch-Schönlein purpura in children. Report of 100 patients and review of the literature. Medicine. (1999) 78:395–409. 10.1097/00005792-199911000-00005 10575422

[40] Mahr A Guillevin L Poissonnet M Aymé S. Prevalences of polyarteritis nodosa, microscopic polyangiitis, Wegener's granulomatosis, and Churg-Strauss syndrome in a French urban multiethnic population in 2000: a capture-recapture estimate. Arthritis Rheum. (2004) 51:92–9. 10.1002/art.20077 14872461

[41] Hatemi G Masatlioglu S Gogus F Ozdogan H. Necrotizing vasculitis associated with familial Mediterranean fever. Am J Med. (2004) 117:516–9. 10.1016/j.amjmed.2004.02.050 15464709

[42] Sachs D Langevitz P Morag B Pras M. Polyarteritis nodosa and familial mediterranean fever. Rheumatology. (1987) 26:139–41. 10.1093/rheumatology/26.2.139 2881591

[43] Glikson M Galun E Schlesinger M Cohen D Haskell L Rubinow A. Polyarteritis nodosa and familial Mediterranean fever: a report of 2 cases and review of the literature. J Rheumatol. (1989) 16:536–9. 2568489

[44] Schlesinger M Oren S Fano M Viskoper JR. Perirenal and renal subcapsular haematoma as presenting symptoms of polyarteritis nodosa. Postgrad Med J. (1989) 65:681–3. 10.1136/pgmj.65.767.681 2575253 PMC2429190

[45] Koçak H Çakar N Hekimoglu B Atakan C Akkök N Ünal S. The coexistence of familial Mediterranean fever and polyarteritis nodosa; report of a case. Pediatr Nephrol. (1996) 10:631–3. 10.1007/s004670050176 8897571

[46] Akpolat T Yilmaz E Ozen S Akpolat I Danaci M Kandemir B. M680I(Arm2)/M694V(Med) mutations in a patient with familial Mediterranean fever and polyarteritis nodosa. Nephrol Dial Transplant. (1998) 13:2633–5. 10.1093/ndt/13.10.2633 9794574

[47] Basaranoglu M Mert A Tabak F Apaydin S Aktuglu Y Ozdogan H. A case of familial Mediterranean fever and polyarteritis nodosa complicated by spontaneous bilateral perirenal and subcapsular splenic haemorrhage. Rheumatology. (1999) 38:794–6. 10.1093/rheumatology/38.8.794 10501441

[48] Korkmaz C Zubaroglu I Kaya T Akçar N Gürbüz E Ozen S. A case of familial Mediterranean fever, Behçet's disease and polyarteritis nodosa complicated by perirenal haematoma. Clin Exp Rheumatol. (2001) 19(5 Suppl. 24):S78–79. 11760409

[49] Ozen S Ben-Chetrit E Bakkaloglu A Gur H Tinaztepe K Calguneri M Polyarteritis nodosa in patients with Familial Mediterranean Fever (FMF): a concomitant disease or a feature of FMF? Semin Arthritis Rheum. (2001) 30:281–7. 10.1053/sarh.2001.19958 11182028

[50] Akar S Goktay Y Akinci B Tekis D Biberoglu K Birlik M. A case of familial Mediterranean fever and polyarteritis nodosa complicated by spontaneous perirenal and subcapsular hepatic hemorrhage requiring multiple arterial embolizations. Rheumatol Int. (2005) 25:60–4. 10.1007/s00296-003-0424-y 14712330

[51] Bakkaloglu SA Muzaç S Akpek S Soylemezoglu O Buyan N Hasanoglu E. Polyarteritis nodosa in a case of familial mediterranean fever. Pediatr Nephrol. (2004) 19:536–8. 10.1007/s00467-003-1390-z 14963762

[52] Calguneri M Apras S Ozbalkan Z Ozturk MA Ertenli I Kiraz S. The efficacy of continuous interferon alpha administration as an adjunctive agent to colchicine-resistant familial mediterranean fever patients. Clin Exp Rheumatol. (2004) 22 (4 Suppl. 34):S41–44. 15515783

[53] Candan F Ayan S Tas F Gökce G Elagoz S. Spontaneous renal laceration as the presenting feature of polyarteritis nodosa in a patient with familial mediterranean fever after hepatitis A infection. Rheumatol Int. (2005) 25:475–7. 10.1007/s00296-005-0597-7 15765217

[54] Standing ASI Eleftheriou D Lachmann HJ Brogan PA. Familial mediterranean fever caused by homozygous E148Q mutation complicated by Budd-Chiari syndrome and polyarteritis nodosa. Rheumatology. (2011) 50:624–6. 10.1093/rheumatology/keq405 21149248

[55] Gur H Eherenfeld M Tchakmakjian L Sidi Y. Polyarteritis Nodosa: a report from Israel. Am J Med Sci. (1999) 317:238–42. 10.1016/S0002-9629(15)40513-0 10210359

[56] Ozen S Saatci U Balkanci F Besbas N Bakkaloglu A Tacal T. Familial mdierranean fever and Polyarteritis nodosa. Scand J Rheumatol. (1992) 21:312–3. 10.3109/03009749209099250 1362008

[57] Baysun S Demircin G Erdoð*an Ö Bülbül M Yildiz YT Öner A Multiple visceral hematomas in a child with familial Mediterranean fever: question. Pediatr Nephrol. (2008) 23:1233–1233. 10.1007/s00467-007-0677-x 18183427

[58] Dollberg L Garti R Geifman M Rosenfeld J. Periarteritis nodosa in a patient with familial Mediterranean fever. Dapim Refuiim Folia Med. (1965) 24:408–15. 4379550

[59] Dor JF Clauvel JP Degos L Mongin F. Spontaneous perirenal haematoma occurring during 1 familial mediterranean fever. 3 cases (author's transl). Nouv Presse Med. (1979) 8:1927–9. 37484

[60] Mongin M Imbert P Dor JF Kasbarian M. Spontaneous renal subcapsular hematoma and periodic disease (apropos of a case). Mars Med. (1972) 109:483–9. 4404688

[61] Tinaztepe K Güçer S Bakkaloglu A Tinaztepe B Familial Mediterranean fever and polyarteritis nodosa: experience of five paediatric cases. A causal relationship or coincidence? Eur J Pediatr. (1997)156:505–6. 9208253

[62] Ertan P Mir S Serdaroglu E Kasirga E Taneli F Kandioglu AR Persistan karin agrisi: familyal akdeniz atesi ve poliarteritis nodosa birlikteligi. Ege Pediatri Bül. (2003) 10:159–61.

[63] Pechère M Helfer C Laurencet FL. Periodic disease and periarteritis nodosa in the same patient: coincidence? Schweiz Med Wochenschr. (1991) 121:1166–8. 1681586

[64] Pagnoux C Seror R Henegar C Mahr A Cohen P Le Guern V. Clinical features and outcomes in 348 patients with polyarteritis nodosa: A systematic retrospective study of patients diagnosed between 1963 and 2005 and entered into the French Vasculitis Study Group Database. Arthritis Rheum. (1963) 62:616–26. 10.1002/art.27240 20112401

[65] Mahr A Belarbi L Wechsler B Jeanneret D Dhote R Fain O. Population-based prevalence study of Behçet's disease: differences by ethnic origin and low variation by age at immigration. Arthritis Rheum. (2008) 58:3951–9. 10.1002/art.24149 19035493

[66] Ben-Chetrit E Cohen R Chajek-Shaul T. Familial mediterranean fever and Behçet's disease–are they associated? J Rheumatol. (2002) 29:530–4. 11908568

[67] Krause I Yankevich A Fraser A Rosner I Mader R Zisman D. Prevalence and clinical aspects of Behcet's disease in the north of Israel. Clin Rheumatol. (2007) 26:555–60. 10.1007/s10067-006-0349-4 16897122

[68] Jaber L Milo G Halpern GJ Krause I Weinberger A. Prevalence of Behçet's disease in an Arab community in Israel. Ann Rheum Dis. (2002) 61:365–6. 10.1136/ard.61.4.365 11874845 PMC1754053

[69] Watad A Tiosano S Yahav D Comaneshter D Shoenfeld Y Cohen AD Behçet's disease? and familial Mediterranean fever: two sides of the same coin or just an association? A cross-sectional study? aEur J Intern Med. (2017) 39:75–8. 10.1016/j.ejim.2016.10.011 27776949

[70] Birlik M Tunca M Hizli N Soytürk M Yeniçerioglu Y Ozcan MA. Coexistence of familial Mediterranean fever with sacroiliitis and Behçet's disease: a rare occurrence. Clin Rheumatol. (1998) 17:397–9. 9805187 10.1007/BF01450901

[71] Bilginer Y Ayaz NA Ozen S. Anti-IL-1 treatment for secondary amyloidosis in an adolescent with FMF and Behçet's disease. Clin Rheumatol. (2010) 29:209–10. 10.1007/s10067-009-1279-8 19774408

[72] Matsuda M Nakamura A Tsuchiya S Yoshida T Horie S Ikeda S. Coexistence of familial mediterranean fever and behçet's disease in a japanese patient. Intern Med. (2006) 45:799–800. 10.2169/internalmedicine.45.1560 16847372

[73] Mobini M. Familial mediterranean fever in an Iranian patient with behcet disease. Casp J Intern Med. (2011) 2:344–6. 24551444 PMC3895835

[74] Schlesinger M Kopolovic J Viskoper RJ Ron N. A case of familial mediterranean fever with cutaneous vasculitis and immune complex nephritis: light, electron, and immunofluorescent study of renal biopsy. Am J Clin Pathol. (1983) 80:511–4. 10.1093/ajcp/80.4.511 6353905

[75] Serrano R Martinez MA Andres A Morales JM Samartin R. Familial mediterranean fever and acute myocardial infarction secondary to coronary vasculitis. Histopathology. (1998) 33:163–7. 10.1046/j.1365-2559.1998.00462.x 9762550

[76] Oguzkurt P Akçören Z Kale G Tanyel F. Polyarteritis nodosa involving the hepatobiliary system in an eight-year-old girl with a previous diagnosis of familial mediterranean fever. Eur J Pediatr Surg. (2000) 10:145–7. 10.1055/s-2008-1072346 10877088

[77] Braun E Azzam ZS Schapira D Guralnik L. Acute vasculitis with multiorgan involvement in a patient with familial mediterranean fever. Am J Med Sci. (2003) 325:363–4. 10.1097/00000441-200306000-00007 12811232

[78] Cefle A Kilicaslan I Gul A Kamali S Inanc M Konice M. Necrotizing crescentic glomerulonephritis with granulomatous vasculitis in a patient with familial Mediterranean fever and renal amyloidosis. Clin Exp Rheumatol. (2004) 22 (4 Suppl. 34):80. 15515794

[79] Cocco G Case report Vasculitis, left bundle branch block, and Raynaud's phenomenon as a manifestation of familial Mediterranean fever. Arch Med Sci. (2009) 4:460–4.

[80] Satoh S Itoh C Nakamura N. A case of frosted branch angiitis associated with retinal vein occlusion as a complication of familial Mediterranean fever. Nippon Ganka Gakkai Zasshi. (2010)114:621–8. 20681258

[81] Zihni FY Kalfa M Ocakçi PT Tarhan F Parildar M Keser G. Coexistence of Takayasu's arteritis with familial mediterranean fever. Rheumatol Int. (2012) 32:1675–8. 10.1007/s00296-011-1853-21416236

[82] Alibaz-Oner F Yilmaz N Can M Direskeneli H. A case of Takayasu's arteritis associated with familial mediterranean fever. Clin Exp Rheumatol. (2012) 30(3 Suppl. 72):S117. 22935091

[83] Zenone T Puget M. Cogan's syndrome in a patient with familial Mediterranean fever. Clin Exp Rheumatol. (2012) 30:141. 22261355

[84] Luger S Harter PN Mittelbronn M Wagner M Foerch C. Brain stem infarction associated with familial Mediterranean fever and central nervous system vasculitis. Clin Exp Rheumatol. (2013) 31 (3 Suppl. 77):93–5. 23710607

[85] Komatsu S Honma M Igawa S Tsuji H Ishida-Yamamoto A Migita K. Cutaneous necrotizing vasculitis as a manifestation of familial Mediterranean fever. J Dermatol. (2014) 41:827–9. 10.1111/1346-8138.12588 25109905

[86] Tekin B Salman A Tuglular S Guler D Ozen G Direskeneli H. Unilateral cutaneous vasculitis: an uncommon presentation and a possible explanation. Indian J Dermatol Venereol Leprol. (2015) 81:518–9. 10.4103/0378-6323.162319 26261133

[87] Ozates S Ozdal PÇ Teke MY. Frosted branch angiitis secondary to familial mediterranean fever resembling central retinal vein occlusion. Case Rep Ophthalmol Med. (2016) 2016:1–4. 10.1155/2016/2916027 28044118 PMC5164904

[88] Ben-Chetrit E Yazici H. Non-thrombocytopenic purpura in familial Mediterranean fever-comorbidity with Henoch-Schönlein purpura or an additional rare manifestation of familial Mediterranean fever? Rheumatol Oxf Engl. (2016) 55:1153–8. 10.1093/rheumatology/kev378 26464521

[89] Fayand A Sarrabay G Belot A Hentgen V Kone-Paut I Grateau G Les multiples facettes du déficit en ADA2, vascularite, maladie auto-inflammatoire et immunodéficit : mise au point à partir des 135 cas de la littérature. Rev Méd Interne. (2018)39:297–306. 10.1016/j.revmed.2017.11.006 29273180

[90] Navon Elkan P Pierce SB Segel R Walsh T Barash J Padeh S. Mutant adenosine deaminase 2 in a polyarteritis nodosa vasculopathy. N Engl J Med. (2014) 370:921–31. 10.1056/NEJMoa1307362 24552285

[91] Direskeneli H. Autoimmunity vs autoinflammation in Behcet's disease: do we oversimplify a complex disorder? Rheumatol Oxf Engl. (2006) 45:1461–5. 10.1093/rheumatology/kel329 16998234

[92] Khairallah M Accorinti M Muccioli C Kahloun R Kempen JH. Epidemiology of Behçet disease. Ocul Immunol Inflamm. (2012) 20:324–35. 10.3109/09273948.2012.723112 23030353

